# Sternal reconstruction using titanium plates for complicated upper hemi-sternum dehiscence

**DOI:** 10.1093/jscr/rjaf566

**Published:** 2025-07-30

**Authors:** Mian Mustafa Kamal, Mohsin Shabbir, Sothagar Subramaniam, Cha Rajakaruna

**Affiliations:** Department of Cardiac Surgery, Bristol Heart Institute, University Hospital Bristol and Weston NHS Foundation Trust, Bristol, United Kingdom; Department of Cardiac Surgery, Bristol Heart Institute, University Hospital Bristol and Weston NHS Foundation Trust, Bristol, United Kingdom; Department of Cardiac Surgery, Bristol Heart Institute, University Hospital Bristol and Weston NHS Foundation Trust, Bristol, United Kingdom; Department of Cardiac Surgery, Bristol Heart Institute, University Hospital Bristol and Weston NHS Foundation Trust, Bristol, United Kingdom

**Keywords:** sternum, plating, rigid fixation

## Abstract

Sternal dehiscence (SD) with or without deep sternal wound infection is one of the troublesome complications of medium sternotomy. It is associated with a significant increase in post-operative morbidity and health care costs. In order to minimize the risk of SD there is a growing trend towards minimally invasive and sternal sparing approaches. Traditionally, SD is surgically managed by rewiring with or without the Robesck technique. However, this approach may not be effective in high-risk patients who are at increased risk of recurrent sternal breakdown. Recently, rigid sternal fixation using titanium plates has evolved as an alternative treatment option in the high-risk cohort. We present surgical management of a complicated upper hemi-sternal dehiscence following aortic valve replacement via upper hemi-sternotomy. We performed reconstruction of the upper hemi-sternum using titanium plates to achieve rigid sternal fixation with excellent results.

## Introduction

Post cardiac surgery sternal dehiscence (SD) with or without deep surgical wound infection (DSWI) is a complication of median sternotomy with a reported incidence of 0.5%–5.0% [1, 2]. It is among the major causes of post-operative morbidity and mortality requiring repeated hospital admissions and increasing health care costs [2].

Median sternotomy is the conventional approach to access the heart and great vessels [1, 3]. However, nowadays minimally invasive approaches are increasingly utilized to avoid complications of full sternotomy [4]. These approaches include hemi-sternotomy, which leaves part of the sternum intact, thus reducing the risk of SD. Hemi-sternotomy though protective, is not an exception to dehiscence as presented in this case. Traditional treatment of SD includes bringing the dehisced sternal edges together by rewiring using stainless steel wires [1, 4]. However, there is an increased risk of recurrent breakdown with this technique especially in high-risk cohort [3]. Recently, rigid sternal fixation using titanium plates is gaining popularity due to its superior results over traditional treatment [1].

We report a complex reconstruction of SD in a high-risk patient who had mini aortic valve replacement via upper hemi-sternotomy. To our knowledge, this is the first case of its kind reported in the literature for upper hemi-sternal dehiscence managed with titanium sternal plates. The report will guide surgeons regarding the surgical technique of rigid sternal fixation in this rare complication.

## Case report

A 69-year-old lady with a body mass index of 43 kg/m^2^ had an elective biological aortic valve replacement via upper hemi-sternotomy. Her post-operative recovery was uneventful. She presented 7 months later to the outpatient clinic with sternal pain and ‘clicking’. She had no signs of infection. Her co-morbidities included hypertension, hypercholesterolemia, and severe osteoarthritis of knees, which limited her mobility. Physical examination revealed a healed sternal wound with palpable sternal clicking.

**Figure 1:**
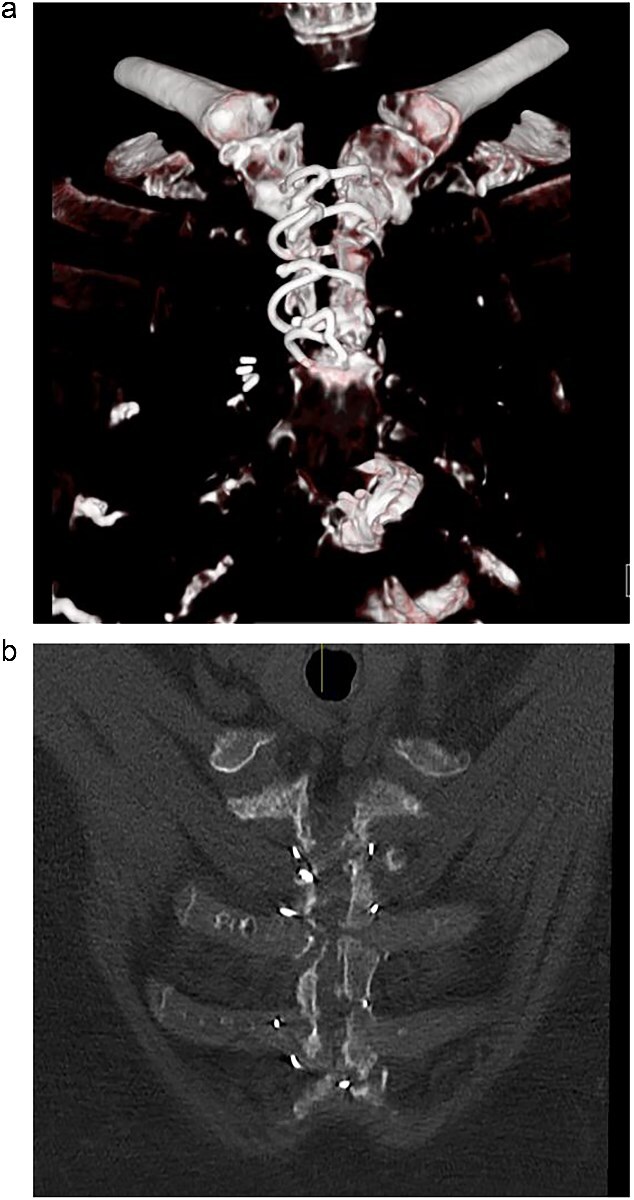
(a) Pre-operative CT scan showing dehisced upper hemi-sternum. (b) Pre-operative CT showing significant bony loss on the right hemi-sternum.

A computed tomography (CT) of the sternum revealed distracted upper sternal edges by 5-11 mm (Fig. 1a). The right upper hemi-sternum had multiple fractures with significant bone loss (Fig. 1b). Her lung fields were clear and baseline bloods were normal. She was diagnosed with sterile mechanical SD and planned for elective rigid sternal fixation.

### Operative technique

**Figure 2:**
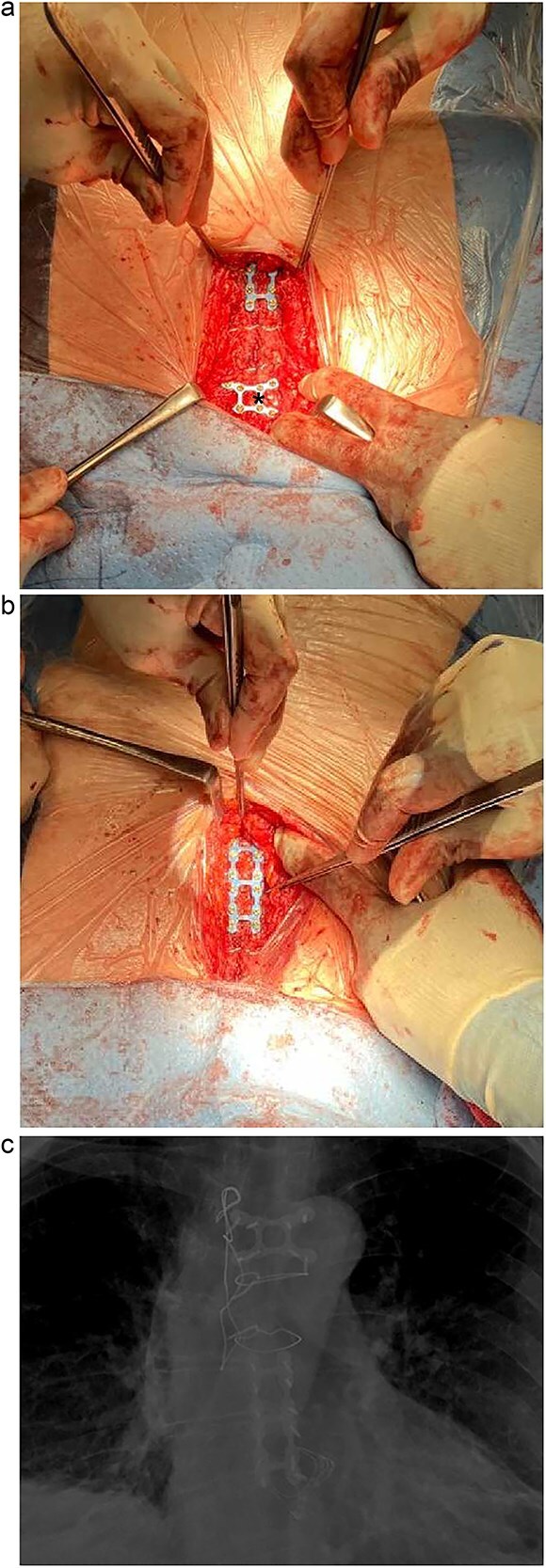
(a) Intra-operative photo demonstrating titanium X-plate (Biomet-8 hole) marked by *. (b) Intra-operative photo demonstrating titanium ladder plate (Biomet-12 hole). (c) Post-operative CXR demonstrating the plated sternum.

The sternum was exposed via a previous upper midline incision and CT findings were noted. Previous placed sternal wires were removed and the sternal edges debrided. Due to significant bony loss a modified Robicsek was done on the right sternal edge. Sternal wall thickness was then measured on each side to help select the size of screws. The sternal edges were then approximated and held reduced with 2 stainless steel sternal wires. Rigid fixation of the manubrium was carried out with titanium X-plate (Biomet-8 hole) (Fig. 2a). A second titanium ladder plate (Biomet-12 hole) was used to fix the upper halves of the sternal edges and stabilize them relative to the intact lower half (Fig. 2b). The titanium plates were fixed on the sternum with self-drilling locking screws. The subcutaneous tissue and skin were closed in layers. Multiple tissue samples were taken and sent to microbiology. She made an uneventful recovery and was discharged home on the third post-operative day. Figure 2c is the post-operative CXR demonstrating the metal work performed.

### Follow up

Tissue samples for microbiology did not isolate any organisms. She was well at follow-up in clinic at 6 weeks and examination revealed a stable sternum and normally healing sternal wound. Further follow-up at 6 and 12 months showed no issues with her sternum.

## Discussion

SD can be sterile or associated with infection known as DSWI. Risk factors for SD include: obesity, heart failure, diabetes, chronic obstructive pulmonary disease, use of bilateral internal mammary arteries, re-operation, and technical operative mistakes [2]. Definitive treatment of SD is surgical re-approximation, which may be preceded by antibiotics and a series of wound debridement if associated with DSWI. In high-risk patients where the risk of recurrent SD is high, rigid sternal fixation is an alternative to traditional sternal rewiring [2]. It is based on the principles of orthopaedic surgery, where sternal edges are approximated and kept fixed relative to each other using various types of titanium plates and screw osteo-synthesis to promote effective healing [2]. The superiority of rigid sternal fixation over conventional techniques of Robicsek or simple rewiring in primary and secondary sternal closure has been demonstrated [1, 2].

Rigid sternal fixation is associated with certain disadvantages. These include risk of infection, cost, availability, difficulty in emergent re-entry, and need for operative expertise [1]. However, modern plating systems now have strategies for emergent re-entry.

Primary rigid sternal fixation (PRSF) is also gaining attention particularly in high-risk patients. However, routine use of PRSF is challenged in the literature, and various types of scoring systems have been introduced to identify high-risk patients for SD and who will benefit with PRSF [5].

We opted for rigid fixation because the patient was at high risk of recurrent SD. In our case, the right hemi-sternum had multiple fractures and severe bone loss, perhaps due to paramedian hemi-sternotomy, which emphasizes the importance of balanced midline sternotomy. Our current practice is to offer primary rigid sternal fixation in high-risk patients.
